# The Phytochemical Properties of Low-Grade Longan Syrup and Its Potential Use as a Dietary Supplement for Honey Bees

**DOI:** 10.3390/insects15120946

**Published:** 2024-11-29

**Authors:** Phurichaya Lertlakkanawat, Sarana Rose Sommano, Khanchai Danmek, Surat Hongsibsong, Chuleui Jung, Saeed Mohamadzade Namin, Malaiporn Wongkaew, Bajaree Chuttong

**Affiliations:** 1 Multidisciplinary Program in Biotechnology, Division of Plant Biotechnology, The Multidisciplinary and Interdisciplinary School, Chiang Mai University, Chiang Mai 50200, Thailand; phurichaya_l@cmu.ac.th; 2 Plant Bioactive Compound Laboratory (BAC), Department of Plant and Soil Sciences, Faculty of Agriculture, Chiang Mai University, Chiang Mai 50200, Thailand; sarana.s@cmu.ac.th (S.R.S.); malaiporn@rmutl.ac.th (M.W.); 3 School of Agriculture and Natural Resources, University of Phayao, Phayao 56000, Thailand; khanchai.da@up.ac.th; 4 School of Health Sciences Research, Research Institute for Health Sciences, Chiang Mai University, Chiang Mai, 50200, Thailand; 5 Environmental, Occupational Health Sciences and Non-Communicable Diseases Center of Excellence, Research Institute for Health Sciences, Chiang Mai University, Chiang Mai, 50200, Thailand; 6 Department of Plant Medicals, Andong National University, Andong 36729, Republic of Korea; cjung@andong.ac.kr; 7 Agricultural Science and Technology Institute, Andong National University, Andong 36729, Republic of Korea; saeedmn2005@gmail.com; 8 Department of Horticulture, College of Agricultural Science, Oregon State University, Corvallis, OR 97331, USA; 9 Program of Food Production and Innovation, Faculty of Integrated Science and Technology, Rajamangala University of Technology Lanna, Chiang Mai 50300, Thailand; 10 Meliponini and Apini Research Laboratory, Department of Entomology and Plant Pathology, Faculty of Agriculture, Chiang Mai University, Chiang Mai 50200, Thailand

**Keywords:** dietary supplement, *Apis mellifera*, lifespan, longan, phytochemical

## Abstract

Climate change significantly affects honey bee populations and their access to natural food sources, demanding alternative economic feed sources. Longan stands out as the most important fruit crop in Southeast Asia, but with a surplus of low-grade fruit that is not suitable for the market. This study investigates the potential of longan syrup as an alternative carbohydrate source for honey bees by measuring sugar composition, phytochemical profiles, feed, and survival, as well as the resulting gut microbial changes. Processed longan syrup contains 8.2, 85.1, and 33.1 mg/g of sucrose, glucose, and fructose. Total phenolic and flavonoid contents were 24.94 and 129.78 mg/g, respectively, showing a 2,2-diphenyl-1-picrylhydrazyl (DPPH) radical inhibition rate of 26.78% and an 87.82% antiradical activity rate via 2,2′-azino-bis (3-ethylbenzothiazoline-6-sulfonic acid) (ABTS). In vitro supplementation studies indicated that survival was highest in the 50% *w*/*v* sucrose syrup condition (control), followed by honey bees fed with 10%, 20%, and 30% longan syrup concentrations, respectively. Analyses of honey bee gut microbiomes revealed that longan syrup supplementation increased fermentative microorganisms such as *Lactobacillus* and *Fructobacillus*, which are beneficial for honey bees’ health. Even though the addition of higher amounts of longan syrup would not be recommended, a 10% addition would be beneficial to honey bees’ health through the modulation of gut microbiomes, demonstrating its potential as a dietary supplement that is a cost-effective and sustainable alternative to sugar syrup during shortages of natural carbohydrate sources.

## Simple Summary

This study aims to explore carbohydrate feed for honey bees from low-grade longan fruit as well as evaluate the phytochemicals in longan syrup as a supplement for honey bees to enhance their health, particularly in terms of survival and gut microbial composition. Findings suggested that lower concentrations of longan syrup are advisable and beneficial for bee health. Additionally, longan syrup supplementation promoted the growth of beneficial fermentative microorganisms, which are advantageous for honey bees.

## 1. Introduction

Honey bees play a crucial role in agriculture, significantly enhancing productivity and economic value through their contribution to pollination and honey production [1]. In Thailand, several species of bees are used in commercial beekeeping, including *Apis mellifera*, *Apis cerana*, and stingless bees. *A. mellifera* dominates beekeeping, representing 89% of total colonies in northern Thailand [2]. Adequate food resources, primarily derived from fruit orchards and wildflowers, are essential for successful beekeeping in the northern regions. In northern Thailand, longan (*Dimocarpus longan*) flowers serve as the primary natural food source for honey bees, as beekeepers predominantly rear bees in longan orchards while supplementing their diet with feeds rich in protein, minerals, and vitamins. Nevertheless, during the period from April to October, when natural food sources are typically limited, beekeepers commonly turn to sugar syrup (mixed with water in ratios of 1:1 or 2:1) as a substitute for carbohydrates [3,4]. Sugar is commonly applied at a rate of approximately 50 kg per hive, leading to an estimated annual cost of around 30 USD [5]. While this approach effectively compensates for the bees’ insufficient food supply, it concurrently raises production costs. As such, it is worthwhile to explore substitute sugar sources obtained from low-cost raw materials.

Longan, a renowned tropical fruit which is native to Southeast Asia, is extensively cultivated in the northern region of Thailand, with major production centers in the Chiang Mai, Lamphun, Chiang Rai, and Lampang provinces, amounting to 160,000 hectares of land and an annual yield exceeding 642,192 tons [6]. Significant losses are observed during the post-harvest and handling stages, particularly during the sorting of fresh fruit. Commercial grades are generally defined by fruit size diameter, including Grade A (>25 mm) and Grade B (22–25 mm), while Grade C (<21 mm) is considered non-marketable, leading to waste in longan orchards [7].

Approximately 15% of the longan’s flesh consists of carbohydrates, specifically sucrose, glucose, and fructose in a 2:1:1 ratio. Interestingly, these sugars serve as the primary food source for honey bees [2,8]. Moreover, longan seeds, pulp, and pericarp contain several important phytochemicals, including polyphenols such as gallic acid, ellagic acid, corilagin, vanillic acid, ethyl gallate, caffeic acid, and *p*-coumaric acid. Additionally, they are rich in flavonoids like flavogallonic acid, kaempferol, epicatechin, and quercetin [9]. These phytochemical components have notable effects on bees’ lifespans, potentially increasing a honey bee’s longevity and enhancing gut microbial diversity and abundance [10]. The microorganisms in the gut provide a wide range of functions that have significant implications for host metabolism and overall health [11]. The gut microbial community has been linked to a variety of traits, including invasive behaviors, nest sanitation, longevity, fecundity, and health [12]. Despite awareness of this, limited research has been conducted on the sugar components and phytochemical profiles of commercially available low-grade longan fruits, as well as their potential application as a feed supplement for honey bees. Therefore, the objectives of this research are to extract and evaluate the sugar composition, perform chemical analysis, and analyze the phytochemical profiles of low-grade longan fruit extracts, with an additional focus on assessing the feasibility of using these fruits as part of a phytochemical diet for honey bees. The hypothesis for this research is that carbohydrates and phytochemicals in longan syrup can improve honey bee health. The anticipated outcome of this research aligns with the zero-waste policy, aiming not only to assist beekeepers in reducing the cost of production but also to sustainably add value to underutilized materials.

## 2. Materials and Methods

### 2.1. Longan Sample Preparation

Fresh low-grade longan fruits of grades B and C (grade B having a diameter between 22 and 25 mm, and grade C comprising fruits with a diameter less than 21 mm) were obtained from local farmers in Hang Dong District, Chiang Mai Province (N18.672776, E98.970770), and Li District, Lamphun Province (N17.930046, E99.001056), in June 2022. The fruits were transported to the laboratory at the Department of Entomology and Plant Pathology, Faculty of Agriculture, Chiang Mai University. The longan fruits were subsequently washed without peeling and then dried in the shade. The juice was extracted using a locally made screw press extractor (2 horsepower and 1.5 kilowatts electric power, with a production capacity of 120 kg per day). A portion of the longan juice was freeze-dried with a freeze-dryer (Martin Christ, Osterode, Germany). The freeze-dried samples were stored in a freezer prior to phytochemical analysis. Another juice portion was boiled in a boiling pot at 90 °C for 10 min to achieve longan syrup after adjusting pH to 5.5 by adding NaOH. Longan syrup was divided into several bottles and stored at 4 °C. This longan syrup was then utilized in the feed supplement experiments for honey bees.

### 2.2. Phytochemical Analysis of Longan Juice

Five grams of the freeze-dried longan juice powder was mixed with 25 mL of water, heated in a water bath at 85 °C for 25 min, then cooled to room temperature, and filtered using a 0.45 µm nylon membrane filter (Whatman, Amersham, UK). This solution was then used for subsequent phytochemical analyses as described in the following sections.

#### 2.2.1. Sugar Composition

Three types of sugar standards, including sucrose, glucose, and fructose, as well as the freeze-dried longan syrup powder were analyzed by high-performance liquid chromatography (HPLC), using the adapted methods described by Yang et al. [13]. The HPLC system was equipped with a diode array detector (Chromaster 5430, Tokyo, Japan) and a column of C18 (250 × 4.6 mm, 5 µm) (HITACHI LaChrom C18 891-5055, Tokyo, Japan). The mobile phase consisted of 50 mM sodium phosphate (pH 6.9) with (A) 15% and (B) 40% acetonitrile (CH_3_CN), with a flow rate of 1.0 mL/min at room temperature, using a gradient elution of 0–8-20% for buffer B with a linear increase from 0 to 10 to 30 min. The wavelength for UV detection was 250 nm.

#### 2.2.2. Total Phenolic Content

This analysis was conducted according to a procedure described previously [14]. The longan solution (30 µL) was mixed with 150 µL of 10% Folin–Ciocalteu reagent. The mixture was stored at room temperature for 6 min. Then, 6% NaHCO_3_ (120 µL) was added. The mixture was thoroughly vortexed for 30 s and stored in a dark place for 60 min. The absorbance of the mixture was measured at 765 nm using a microplate spectrophotometer (SPECTROstar^®^ Nano, BMG LABTECH, Cary, NC, USA). Total phenolic content was expressed as mg gallic acid equivalents (GAEs) per g of dry matter of the longan juice sample.

#### 2.2.3. Total Flavonoid Content

The longan sample solution (25 µL) was mixed with 125 µL of distilled water, followed by the addition of 75 µL of 5% NaNO_2_, and was allowed to stand for 5 min. To the mixture, 15 µL of 10% AlCl_3_ was added and left to stand for 6 min, followed by the addition of 15 µL of 1 M NaOH and 27.5 µL of distilled water. The absorbance of the mixture was evaluated at 510 nm using the microplate spectrophotometer (SPECTROstar^®^ Nano, BMG LABTECH, Cary, NC, USA). Total flavonoid content was illustrated as mg catechin equivalents (QEs) per g of dry matter of the longan juice sample [14].

#### 2.2.4. Antioxidant Activities

1. 2,2-Diphenyl-1-picrylhydrazyl (DPPH) Radical Scavenging Activity

The longan sample solution (25 µL) was combined with 250 µL of 0.2 mM DPPH. Subsequently, the mixture was stirred and allowed to incubate in a dark environment at room temperature for 30 min. The absorbance of the mixture was determined at 517 nm using the microplate reader [14]. The scavenging activity was presented as %DPPH radical inhibition and trolox equivalent mg/g sample. The %DPPH radical inhibition was calculated using the following equation.

%DPPH radical inhibition = [(A_control_ − A_sample_)/A_control_] × 100 (1)

A_control_ is the absorbance of DPPH radical mixed with methanol.

A_sample_ is the absorbance of DPPH radical mixed with the sample.

2. Total Antioxidant Activity by 2,2′-azino-bis (3-ethylbenzothiazoline-6-sulfonic acid) (ABTS)

The longan sample solution (10 µL) was combined with 200 µL of ABTS working solution. Then, the mixture was thoroughly mixed and allowed to stand for 30 min. The absorbance of the mixture was measured at 734 nm using the microplate reader [14]. The working solution was prepared by two stock solutions including 7.0 mM ABTS solution and 2.45 mM K_2_S_2_O_8_ in a 1:1 ratio and kept in a dark place for 12–16 h. The ABTS solution was diluted with PBS pH 7.4 until an absorbance of 0.70 ± 0.02 at 734 nm was obtained. The activity, expressed as %antiradical activity and trolox equivalent mg/g sample, was calculated using the following equation.

%Antiradical activity = [(A_control_ − A_sample_)/A_control_] × 100 (2)

A_control_ is the absorbance of ABTS radical mixed with methanol.

A_sample_ is the absorbance of ABTS radical mixed with the sample.

#### 2.2.5. Qualitative Analysis of Phytochemical Profiles

Five standards in concentrations of 100, 150, 500, and 1000 ppm (gallic acid, catechin, *p*-coumaric acid, vanillic acid, and kaempferol) and the freeze-dried longan syrup powder were analyzed using high-performance liquid chromatography analysis (HPLC) (Hitachi Chromaster, Tokyo, Japan) adapted from the method of Somjai et al. [15]. The samples were diluted in 95% methanol to obtain a concentration between 100 and 1000 µg/mL and then filtrated through a 0.20 µm membrane filter. HPLC was operated with an automatic injection (5210 Autosampler), pump (Chromaster 5110, Tokyo, Japan), and automatic control (Chromaster 5210, Tokyo, Japan). Reverse-phased column chromatography was performed using an Ultra Aqueous C18 (250 × 4.6 mm, 5 µm) (HITACHI LaChrom C18 891-5055, Tokyo, Japan). The mobile phase solution was prepared using mixtures A and B, with mixture A containing formic acid and distilled water in a proportion of 0.4:99.6 [16] and mixture B containing acetonitrile (CH_3_CN), distilled water, and formic acid in a proportion of 85:14.6:0.4 (*v*/*v*), at a flow rate of 1 mL/min and an injection volume of 10 µL [17]. The initial condition was 60% A for 3 min, which was decreased to 25% A for 5 min, with 95% A held for 1 min and then decreased to 80% A for 0.1 min before returning to 80% A for 3.9 min. The total run time was 12 min per sample. Chromatograms were recorded by ultraviolet (UV) detection at 280 nm.

### 2.3. Feeding Experiments of Longan Syrup to Honey Bees

#### 2.3.1. Consumption and Survival

Capped brood combs of the honey bee *Apis mellifera* were collected in October 2022 at the Faculty of Agriculture, Chiang Mai University (N18.793187, E98.961042). Brood combs were incubated overnight at 33 °C and 60% humidity in an incubator. Newly emerged worker bees were collected, and 25 workers were randomly assigned evenly to four groups, with a cage (90 × 110 mm sized cage with three ∅15 mm sized feeding holes) for each group. Five replications were prepared for each treatment. The cages were maintained in a dark environment at 30 °C with 60% humidity in an incubator.

Newly emerged bees were acclimatized to cages by initially being fed sucrose syrup for a 2-day period, followed by subsequent treatments [18]. The treatments were 50% sucrose syrup (*w*/*v*), equivalent to 50 °Brix as a control (SU), and then longan syrup of 10 (LS10), 20 (LS20), and 30% (LS30) concentrations, adjusted to 50 °Brix with a sucrose syrup addition, provided using a 3 mL syringe. All cages were provided with ad libitum access to water, pollen patty (bee pollen mixed with 50% sucrose syrup (*w*/*v*) in a ratio 2:1), and a carbohydrate source. Solution feeders were created by cutting a hole on the top of a 3 mL syringe tube. Pollen was affixed to the bottom of a 1.5 micro-centrifuge tube, renewed every 3 days. Food consumption was recorded daily. Bee mortality was recorded daily for 7 weeks, and deceased bees were consistently removed from the cages [19].

#### 2.3.2. Gut Microbiome Analysis

1. Bee Preparation

The same sets of treatment used in the Consumption and Survival Section, with 5 replications, were prepared under the same conditions. Then, 5 worker bees were sampled from each treatment at 3, 6, 9, 12, 15, and 18 days. Bee samples were preserved in 95% ethanol inside 50 mL Falcon tubes at −20 °C until DNA extraction [20,21]. Before DNA extraction, each bee was individually surface-sterilized with a 90% ethanol solution for 30 s, followed by a wash in sterile water [22]. At each specified time point for each treatment, the entire guts (midgut–hindgut) of 5 bees from 5 replicates were dissected using sterile micro-scissors and forceps [23]. The gut was easily extracted by gently pulling on the sting shaft [24].

2. DNA Extraction

DNA was extracted using the QIAamp DNA Mini Kit (QIAGEN, Hilden, Germany), and the quantity and quality of the DNA extracts were assessed using a Micro UV-Vis Spectrophotometer (Life Sciences, Zhengzhou, China). Purification was carried out using the PCR inhibitor removal kit (Zymo Research, OneStep PCR Inhibitor Removal). The extracted DNA samples were stored at −20 °C before library preparation [25]. Two-step PCR was used for library preparation of the V4 region of 16S rRNA using the primers 515F (5′-GTGCCAGCMGCCGCGGTAA-3′) and 806R (5′-GGACTACHVGGGTWTCTAAT-3′), followed by sequencing using an Illumina MiSeq (Macrogen, Seoul, Republic of Korea). All sequences have been deposited in NCBI’s Sequence Read Archive under the number HN00202979.

3. DNA Sequence Analysis

The initial quality assessment of raw paired-end reads was conducted using FastQC (Babraham Bioinformatics, Cambridge, UK). Following this, the raw sequences were imported into Qiime2 for further analysis. The DADA2 algorithm was employed, which involved trimming the forward reads to 280 bp and the reverse reads to 220 bp to ensure high-quality reads with a Phred quality score of at least 20, assembling the paired-end reads, and detecting chimeras. The Amplicon Sequence Variants (ASVs) were subsequently classified using the SILVA v132 database for taxonomy assignment [25].

### 2.4. Statistical Analysis

The statistical significance of phytochemical analysis was compared using a *t*-test, with a *p*-value < 0.05 considered statistically significant. Mean differences in the consumption rate and the lifespan test in the research data were compared using one-way analysis of variance (ANOVA) followed by Duncan’s Multiple Range Test. Statistical analyses were conducted with SPSS 23.0 software (SPSS Inc., Chicago, IL, USA), with a *p*-value < 0.05 considered statistically significant. The Kaplan–Meier survival analysis (KM) was performed to evaluate the differences in the lifespan of honey bees, using SPSS Version 23. Principal component analysis (PCA) was also conducted to visualize the relationships of gut bacterial taxa richness and diversity between the gut samples from honey bees treated with different carbohydrate resources, using the “FactoMineR” package in R 4.2. All experiments were conducted with 5 replicates for each test.

## 3. Results

### 3.1. Phytochemical Analysis of Longan Juice

The extracted longan juice had a percentage yield of 43.3% and a 11.7 ± 0.69 °Brix with a pH of 3.79 ± 0.02. The sugar used consisted of 3.9, 3.0, and 1.3 g/100 g of sucrose, glucose, and fructose, respectively. Total phenolic and flavonoid contents were 21.68 ± 2.47 and 109.45 ± 10.58 mg/g, respectively, equivalent to 1.01 mg/g gallic acid (GA) and 1.82 mg/g of catechin. The antioxidant activities of DPPH and ABTS were 29.51% and 83.02%, respectively. No other phytochemicals were detected, including caffeic acid, *p*-coumaric acid, vanillic acid, and quercetin (Table 1).

After longan syrup was processed, the results indicated a percentage yield of 6.7% after boiling, which was 15.3 ± 0.06 °Brix with a pH of 5.53 ± 0.02. The sugar used was composed of 0.8, 8.5, and 3.3 g/100 g of sucrose, glucose, and fructose, respectively. Total phenolic and flavonoid contents were 24.94 ± 2.38 and 129.78 ± 14.63 mg/g, respectively, equivalent to 1.45 mg/g of gallic acid (GA) and 1.41 mg/g of catechin. The antioxidant activities of DPPH and ABTS reached 26.78% and 87.82%, respectively. No other phytochemicals were detected, including caffeic acid, *p*-coumaric acid, vanillic acid, and quercetin. However, statistically significant differences were observed between longan juice and longan syrup in terms of sugar composition and catechin content (Table 1).

### 3.2. Evaluation of Low-Grade Longan Fruits as Part of a Phytochemical Diet for Honey Bees

#### 3.2.1. Consumption Rate of Longan Supplements

Consumption by honey bees was 16.10 ± 6.14, 14.17 ± 4.77, 10.33 ± 4.17, and 9.73 ± 3.00 µL/bee/day in the groups of honey bees fed with 50% *w*/*v* sucrose syrup (control), 10%, 20%, and 30% longan syrup, respectively (Table 2). The results indicate that the lowest consumption rate was observed in the group of honey bees fed with the highest concentration of longan syrup; however, no significant differences were observed between the consumption rates of different treatments.

#### 3.2.2. Lifespan of Honey Bees

**Figure 1:**
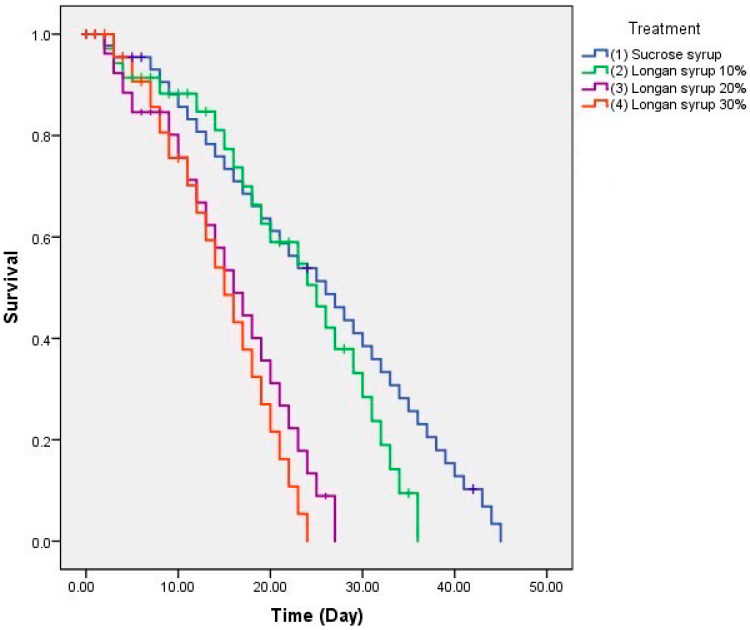
Lifespan of honey bees fed with different concentrations of longan syrup: (1) 50% *w*/*v* sucrose syrup; (2) 10% longan syrup; (3) 20% longan syrup; and (4) 30% longan syrup.

The results of this study showed that honey bees fed with 50% *w*/*v* sucrose syrup (control) had the highest average longevity of 25.3 ± 5.51, followed by longan syrup at concentrations of 10%, 20%, and 30%, which resulted in longevities of 23.0 ± 5.69, 15.8 ± 3.54, and 14.8 ± 3.60 days, respectively. Median longevity showed the same trend (Table 2, Figure 1).

#### 3.2.3. Gut Microbiome Diversity of Honey Bees

**Figure 2:**
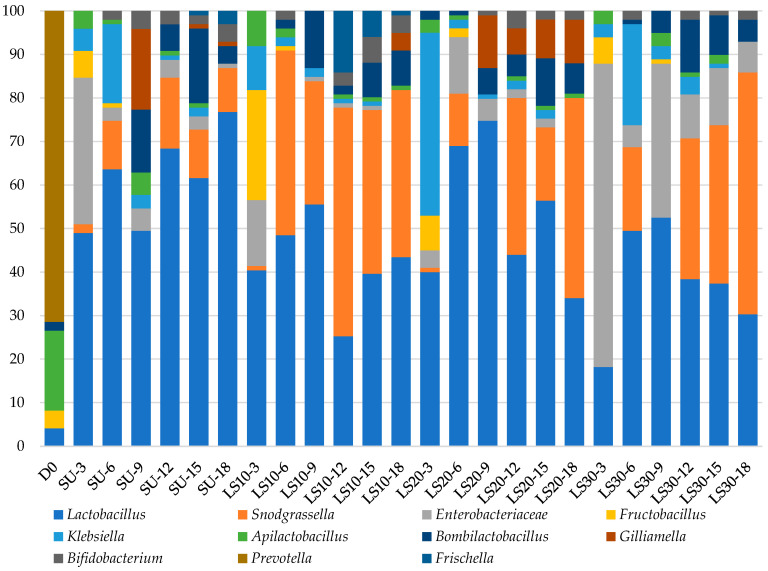
Taxonomic trends of bacterial communities among treatments over time (D0 = day 0; SU-3, SU-6, SU-9, SU-12, SU-15, and SU-18 = sucrose syrup on days 3–18; LS10-3, LS10-6, LS10-9, LS10-12, LS10-15, and LS10-18 = 10% longan syrup on days 3–18; LS20-3, LS20-6, LS20-9, LS20-12, LS20-15, and LS20-18 = 20% longan syrup on days 3–18; LS30-3, LS30-6, LS30-9, LS30-12, LS30-15, and LS30-18 = 30% longan syrup on days 3–18).

A gut microbiome analysis of honey bees was conducted for each treatment in different intervals, and the core bacterial communities for each treatment are shown in Figure 2. The 18 major bacterial taxonomic units that were observed included *Lactobacillus*, *Snodgrassella*, *Enterobacteriaceae*, *Fructobacillus*, *Klebsiella*, *Apilactobacillus*, *Bombilactobacillus*, *Gilliamella*, *Bifidobacterium*, *Prevotella*, and *Frischella*.

Based on the taxonomic trends of bacterial communities (Figure 2), it was found that the predominant microorganisms on day 0 (D0) were *Lactobacillus*, *Fructobacillus*, *Apilactobacillus*, *Bombilactobacillus*, and *Prevotella*. In this group, *Prevotella* and *Apilactobacillus* were the most dominant microorganisms, respectively, which are commonly found in the gut of honey bees [11,26]. The results showed a decreasing tendency of bacterial diversity when honey bees were fed sucrose syrup (SU) from days 3 to 18, while the proportion of *Lactobacillus*, *Enterobacteriaceae*, *Snodgrassella*, and *Bombilactobacillus* increased.

When honey bees were fed 10% longan syrup, there was an increase in *Lactobacillus*, *Enterobacteriaceae*, *Fructobacillus*, *Klebsiella*, and *Apilactobacillus* on day 3. However, from days 6 to 18, *Lactobacillus*, *Snodgrassella*, and *Bombilactobacillus* increased, while *Enterobacteriaceae* decreased. When honey bees were fed 20% longan syrup, *Lactobacillus*, *Klebsiella*, and *Fructobacillus* increased on day 3. From days 6 to 18, there was a significant increase in the proportion of *Lactobacillus*, *Snodgrassella*, *Bombilactobacillus*, and *Gilliamella*, while *Enterobacteriaceae* decreased. Moreover, the results showed that feeding honey bees with 30% longan syrup led to an increasing trend for *Lactobacillus*, *Snodgrassella*, and *Enterobacteriaceae*.

**Figure 3:**
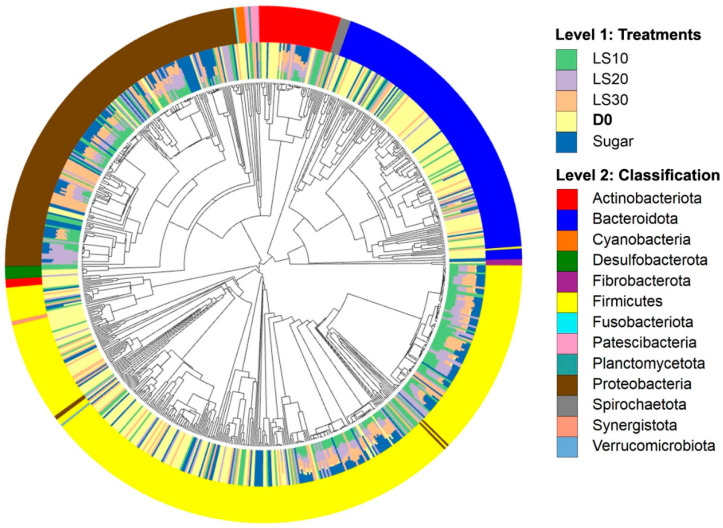
The circular phylogenetic tree and corresponding bar chart of gut microbiome composition of honey bees treated with different concentrations. The outer ring (Level 1: Treatments) color codes represent the different treatment groups. The inner ring (Level 2: Classification) shows the classification of the gut microbiome at the phylum level, with each color representing a different phylum.

*Prevotella* (Bacteroidota phyla) also represents a group of microorganisms that could be found in the gut of honey bees on day 0 (D0) (Figure 3). The cluster analysis resulted in two main groups: the control group, which showed a high amount of *Prevotella*, and the remaining groups, which were further subdivided based on their levels of *Prevotella* and *Gillamella*. In particular, bees with a high amount of *Gillamella* were those fed on longan syrup at concentrations ranging from 10% to 20%. The presence of *Prevotella* decreased when bees were fed with longan syrup supplements. In addition, *Bombilactobacillus*, which was less common on day 0, become more prevalent in bees fed with sucrose syrup (SU) on days 9 to 15. Moreover, feeding longan syrup supplements to honey bees did not significantly increase the diversity of microorganisms in the bees’ digestive tracts. However, it did increase fermentative microorganisms such as *Lactobacillus* across all longan syrup concentrations. Additionally, *Snodgrassella* and *Frischella*, which are among normal microorganisms in honey bees, also increased. *Enterobacteriaceae*, another group of microorganisms, increased when bees were fed longan syrup at the highest concentration (30%).

The circular phylogenetic tree and the corresponding bar chart (Figure 3) illustrate the gut microbiome composition of honey bees treated with different concentrations of longan syrup (LS10, LS20, LS30) or sugar syrup (SU). The phylogenetic tree highlights the diversity and abundance of various microbial taxa present in the gut of honey bees across different treatments. Notably, the Firmicutes and Proteobacteria phyla dominate the microbiome across all treatment groups, as indicated by the prominent yellow and brown sections in the inner ring. The variation in the microbiome composition across different treatments is visually represented by the distribution of colors in the outer ring. This comprehensive visualization provides insights into the microbial community structure and the potential impact of different carbohydrate sources on the gut microbiome of honey bees.

**Figure 4:**
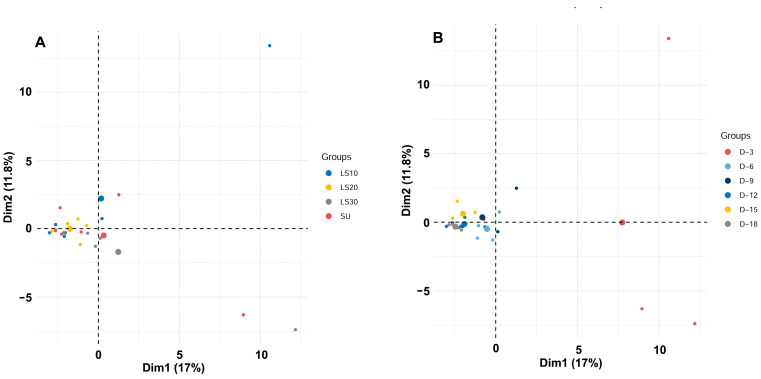
Principal component analysis (PCA) representing the gut microbiome bacterial taxa and their abundances in the honey bee samples fed with different carbohydrate resources. (**A**) Different concentrations of longan syrup; (**B**) date of sampling.

PCA was conducted to understand the relationship between the gut microbiome compositions of honey bees treated with sugar syrup (SU) or different concentrations of longan syrup (LS10, LS20, LS30). The PCA plot (Figure 4A) shows that the first two principal components explain 28.8% of the total variance (17% by Dim1 and 11.8% by Dim2). The results reveal no distinct clustering of gut samples based on the carbohydrate resource treatment, suggesting that the microbiome composition is not significantly affected by the type of carbohydrate provided. In contrast, the PCA plot (Figure 4B) illustrates the gut microbiome composition taking into consideration the date of sampling. It is evident that most samples collected on day 3 (D-3) are positioned on the right side of the graph, indicating a unique microbiome profile at this time point. Conversely, samples collected on later dates (D-6, D-9, D-12, D-15, and D-18) cluster closely together on the left side of the graph. This pattern suggests that the gut microbiome composition of honey bees stabilizes after the initial days of treatment, showing less variation over time. These observations imply that while the type of carbohydrate does not significantly impact the gut microbiome composition, the sampling date plays a crucial role, with early samples showing greater variability.

## 4. Discussion

This study demonstrated that low-grade longan could be used as a carbohydrate food source for honey bees. Longan syrup contains high levels of sucrose, glucose, and fructose, indicating an increased glucose and fructose ratio with significant antioxidant capacity. This is consistent with our findings and those of Surin et al. [27], suggesting that heating elevates reducing sugars. Honey bees cannot directly digest sucrose, as it needs to be inverted into glucose and fructose [28,29]. Additionally, longan syrup has a higher proportion of glucose and fructose, making it easier for bees to digest and benefiting their digestive system. Longan syrup has high antioxidant capacity, but honey bees exhibit increased longevity when fed low concentrations of longan syrup compared with high concentrations. In addition, high levels of phenolics and flavonoids from longan peels and seeds can negatively impact honey bee immunity and metabolism, leading to reduced lifespan [10]. Conversely, higher concentrations of longan syrup result in a lower consumption rate, causing the bees to receive insufficient energy to meet their needs.

However, a small amount of added longan syrup could enhance honey bee health, possibly by modulating gut microbials. The gut microbial community has been linked to a variety of traits, including invasive behaviors, nest sanitation, longevity, fecundity, and health [12]. Based on the results of gut microbiome analyses, *Lactobacillus*, *Snodgrassella*, *Klebsiella*, *Fructobacillus*, and *Enterobacteriaceae* were the predominant bacteria found in the guts of honey bees provided with different diets, and most of these are commonly found in the guts of bees [11,26]. Among them, *Lactobacillus* was the dominant bacterial genus inside the gut. The results from Geldert, Abdo, Stewart, and HS [23] also revealed a significant increase in *Lactobacillus* proliferation when honey bees were solely fed sucrose, consistent with prior research highlighting *Lactobacillus* species as the dominant genus, with a preference for metabolizing sucrose. *Snodgrassella* was another core bacterial category in all of the treated groups, but they are more dominant in the groups fed with longan syrup. *Enterobacteriaceae* were recorded as one of the main bacteria which were highly abundant in the group of honey bees fed with longan 30%, and it seems likely that *Enterobacteriaceae* are highly correlated with the consumption of longan syrup. In addition, Geldert et al. [23] reported that feeding honey bees with phytochemical supplements can result in the presence of *Enterobacteriaceae* that are non-specific members of the microbial community, along with their pronounced negative correlations with lactobacilli. This might indicate that these species can thrive when the specific bacterial community is perturbed and fewer *Lactobacillus* spp. are present [30]. The study results showed the presence of *Klebsiella* and *Fructobacillus*, which are not typically reported as normal microorganisms in the gut of honey bees and which can be correlated with the consumption of longan syrup supplements, since an increased proportion of *Klebsiella* was observed in honey bees fed with higher concentrations of longan syrup. Galatiuk et al. [31] found that *Klebsiella* can cause infectious diseases in bees. Moreover, *Fructobacillus*, which is a part of the Lactic Acid Bacteria (LAB) group, can ferment fructose, helping to improve bees’ immunity [32]. *Bombilactobacillus*, which is known to be affected by some plant protection chemicals, such as antibiotics and weed killers, was also present in all treated groups [32].

On day 0, a specific group of microorganisms, namely *Prevotella*, could be found in the guts of honey bees. *Prevotella* are non-cellulolytic carbohydrate-degrading bacteria that facilitate the digestion of cell wall polysaccharides like xylan [33]. However, the presence of *Prevotella* decreased when honey bees were fed longan syrup supplements. Additionally, *Bombilactobacillus* could be found in the guts of honey bees. It was less common on day 0 but became more prevalent when honey bees were fed sucrose syrup (control) on days 9 and 15. The shift in the gut microbiome composition suggests that dietary changes significantly impact the microbial communities within honey bees. Feeding honey bees with different concentrations of longan syrup appears to influence the prevalence of specific microorganisms. For instance, while longan syrup supplements led to a reduction in *Prevotella*, they also fostered the growth of other beneficial microbes.

Furthermore, these findings indicate that dietary composition significantly impacts the gut microbiome of honey bees. The presence and dominance of specific bacteria like *Lactobacillus*, *Snodgrassella*, and *Enterobacteriaceae* highlight the adaptability of the gut microbiome to different carbohydrate sources. This emphasizes the importance of considering the type and concentration of dietary supplements, such as longan syrup, to promote a healthy and balanced gut microbiome in honey bees.

Understanding these dynamics is crucial for developing dietary strategies to enhance the health and longevity of honey bees. Overall, these findings underscore the importance of understanding the effects of dietary supplements on the gut microbiome of honey bees. The introduction of longan syrup alters the balance of gut microorganisms, with both beneficial and potentially harmful effects. Future research should continue to explore the optimal dietary conditions that support the health and longevity of honey bees, taking into consideration the complex interactions within their gut microbiomes. Moreover, longan seed and peel should be removed before making syrup for honey bees. Longan may contain other undetected phytochemical compounds that could be toxic to honey bees. High levels of phenolics and flavonoids from longan peels and seeds can negatively impact honey bee immunity and metabolism, potentially leading to a reduced lifespan. In this experiment, only adult bees were examined for their lifespans, consumption rates, and microbials. Additional research should focus on bee broods from carbon sources to gain a more comprehensive understanding of their development.

## 5. Conclusions

Our chemical analyses and phytochemical profiles of low-grade longan fruit extracts and longan syrup indicated longan fruit’s suitability as a supplement for honey bees due to its high content of reducing sugars (glucose and fructose), which honey bees can easily digest. Longan syrup also exhibits elevated levels of total phenolics, flavonoids, and antioxidant activities, potentially extending the longevity of honey bees. The lifespan and the mean survival time were highest in the 50% *w*/*v* sucrose syrup (control), followed by honey bees fed with 10%, 20%, and 30% longan syrup concentrations, respectively. It is advisable that the maximum concentration of longan syrup does not exceed 10% to ensure optimal benefits without adverse effects. Regarding the diversity of the gut microbiome in honey bees, supplementation with longan syrup can enhance fermentative microorganisms such as *Lactobacillus* and *Fructobacillus*. However, higher concentrations of longan syrup have been observed to increase *Enterobacteriaceae* levels, which could pose risks to bee health. In addition, the potential effects of phytochemicals should be carefully considered when using them as dietary supplements for honey bees. In conclusion, longan syrup represents a promising dietary alternative for honey bees, offering a sustainable solution to reduce costs and to ensure feed security while promoting honey bees’ health and longevity. Therefore, longan syrup is suited as a supplement feed for honey bees.

## Figures and Tables

**Table 1:** Total soluble solid, pH, and % yield in longan sample preparation and chemical constituents in longan juice and longan syrup.

		Sample		*p*-Value
		Longan Juice	Longan Syrup	
% Yield		43.33	6.67	-
Total soluble solid (°Brix)		11.7 ± 0.69	15.33 ± 0.06	-
pH		3.79 ± 0.02	5.53 ± 0.02	-
Sugar composition (g/100 g)	Sucrose	3.90 *	0.82 ± 0.44 *	0.003
	Glucose	3.01 *	8.51 ± 0.13 *	0.005
	Fructose	1.31 *	3.31 ± 0.01 *	0.013
Total phenolic (mg/g)		21.68 ± 2.47	24.94 ± 2.38	0.260
Total flavonoid (mg/g)		109.45 ± 10.58	129.78 ± 14.63	0.297
Antioxidant activities	DPPH radical inhibition (%)	29.51 ± 6.26	26.78 ± 2.94	0.657
	Antiradical activity (%)	83.02 ± 6.39	87.82 ± 1.79	0.322
Phytochemical profiles	Gallic acid (mg GA/g dry sample)	1.01 ± 0.37	1.45 ± 0.08	0.280
	Catechin (mg CAT/g dry sample)	1.82 ± 0.34 *	1.41 ± 0.53 *	0.032

* This sign indicates statistical significance at the 95% confidence level (*p* ≤ 0.05) in the *t*-test (within rows). The data are average ± SD.

**Table 2:** Consumption rate, mean survival time, and median survival time of honey bees fed with longan syrup in different concentrations, with sucrose as a control.

Treatment	Consumption (µL/Bee/Day) *	Mean Longevity (Days) *	Median Longevity (Days) **
control (SU)	16.10 ± 6.14	25.25 ± 5.51 ^a^	26.00 (15.00–36.00)
10% (LS10)	14.17 ± 4.77	22.95 ± 5.69 ^ab^	25.00 (16.00–31.00)
20% (LS20)	10.33 ± 4.17	15.81 ± 3.54 ^bc^	16.00 (11.00–22.00)
30% (LS30)	9.73 ± 3.00	14.81 ± 3.60 ^c^	15.00 (11.00–20.00)

* The data are average ± SD. ** Median longevity is presented as days (upper and lower interquartile range). Different letters in each column indicate significant differences among the treatments.

## Data Availability

All sequence information has been deposited in the National Center for Biotechnology Information (NCBI) Sequence Read Archive (project number PRJNA1181032).
